# Ankylosing spondylitis: A challenge to anaesthesiologists due to difficulties in airway management and systemic involvement of disease

**DOI:** 10.4103/0019-5049.60507

**Published:** 2010

**Authors:** Anand T Talikoti, K Dinesh, Anand Kumar, Goolappa

**Affiliations:** Department of Anaesthesia and Critical Care, Sri Devaraj Urs Academy of Higher Education, Deemed University, Kolar, India

Sir,

We report a case of ankylosing spondylitis with hepatic disease, with gangrene right leg, posted for right hip disarticulation. A 50-year-old male patient presented with gangrene of right leg up to mid portion. The patient had a fixed spine with absent lumbar and thoracic curvatures. The patient had icterus with slurred speech and was drowsy, but obeyed oral commands. On auscultation of lungs, patients had bilateral basal crepitations and diffuse rhonchi. The x-ray of cervical spine revealed loss of cervical lordosis with ankylosis of apophyseal joints. X-ray of lumbar spine revealed:

- bridging osteophytes over lumbar vertebrae.
- ankylosis of both sacroiliac joints.
- sacralisation of L_5_

Chest x-ray revealed prominent bronchovascular markings with minimal pleural effusion on right side. Lab investigations revealed elevated liver enzymes with raised bilirubin but normal coagulation profile. The risk of surgery and anaesthetic problems were explained to patient and his caretakers and the patient was accepted for anaesthesia under ASA Grade IV E physical status. Consent for emergency tracheostomy was also taken.

The patient was shifted to the operation theatre and intravenous access was obtained with 18 G vasofix; patient was preloaded with 1000 ml of NS while pulse oximeter, NIBP and ECG monitors were connected. Difficult intubation carts, including LMA, bougie, fibre optic bronchoscopy, cricothyrotomy needle were kept ready. The tracheostomy set was also kept ready. The patient was administered spinal anaesthesia in lateral position by paramedian approach with injection bupivacaine heavy (0.5%) 2 ml. The onset of action was delayed with sensory block up to T12 achieved in 18 minutes. Oxygen by mask was administered throughout the surgery. Injection mannitol 20% 100 ml was administered intravenously as prophylaxis against hepatorenal syndrome. Haemodynamic parameters were within normal limits throughout surgery. Duration of sensory block lasted 14 minutes. Postoperative pain relief was given by injection fentanyl 40 μg intravenous followed by intravenous fentanyl infusion at rate of 30 μg/hour titrated to consciousness level of patient.

Our patient had both ankylosing spondylitis and hepatic dysfunction. Since the patient had normal coagulation status, spinal anaesthesia was the contemplated technique. Midline placement of spinal needle is difficult in patients with ankylosing spondylitis due to ossification of interspinous ligaments and bony bridges.[1] Spinal anaesthesia by paramedian approach has been used for total hip replacement surgery in ankylosing spondylitis cases[2,3] Caudal epidural anaesthesia has also been used for hip replacement surgery in ankylosing spondylitis.[4]

Spinal anaesthesia-related fall in mean arterial pressures causes decrease in hepatic blood flow. This point was also kept in mind while administering spinal anaesthesia. Limit on the total dose of drug and correct positioning with head end elevation by 5 degrees achieved adequate level of blockade. The pharmacokinetics of fentanyl is predictable in patients with liver disease.[5]

Spinal anaesthesia by paramedian approach can be a useful technique in patients with ankylosing spondylitis and hepatic dysfunction with normal coagulation status. However, every effort towards maintaining airway and equipment should be made.
